# Characteristic Aspects of Uranium(VI) Adsorption Utilizing Nano-Silica/Chitosan from Wastewater Solution

**DOI:** 10.3390/nano12213866

**Published:** 2022-11-02

**Authors:** Ahmed K. Sakr, Mostafa M. Abdel Aal, Khaled A. Abd El-Rahem, Eman M. Allam, Samia M. Abdel Dayem, Emad A. Elshehy, Mohamed Y. Hanfi, Mohammed S. Alqahtani, Mohamed F. Cheira

**Affiliations:** 1 Department of Civil and Environmental Engineering, Wayne State University, 5050 Anthony Wayne Drive, Detroit, MI 48202, USA; 2 Nuclear Materials Authority, El Maadi, Cairo 11381, Egypt; 3 Department of Chemistry, Faculty of Science, Al-Azhar University, Cairo 11651, Egypt; 4 Institute of Physics and Technology, Ural Federal University, St. Mira, 19, 620002 Yekaterinburg, Russia; 5 Department of Radiological Sciences, College of Applied Medical Sciences, King Khalid University, Abha 61421, Saudi Arabia; 6 BioImaging Unit, Space Research Centre, University of Leicester, Michael Atiyah Building, Leicester LE1 7RH, UK

**Keywords:** chitosan, silicon dioxide, sorption, uranium, kinetics

## Abstract

A new nano-silica/chitosan (SiO_2_/CS) sorbent was created using a wet process to eliminate uranium(VI) from its solution. Measurements using BET, XRD, EDX, SEM, and FTIR were utilized to analyze the production of SiO_2_/CS. The adsorption progressions were carried out by pH, SiO_2_/CS dose, temperature, sorbing time, and U(VI) concentration measurements. The optimal condition for U(VI) sorption (165 mg/g) was found to be pH 3.5, 60 mg SiO_2_/CS, for 50 min of sorbing time, and 200 mg/L U(VI). Both the second-order sorption kinetics and Langmuir adsorption model were observed to be obeyed by the ability of SiO_2_/CS to eradicate U(VI). Thermodynamically, the sorption strategy was a spontaneous reaction and exothermic. According to the findings, SiO_2_/CS had the potential to serve as an effectual sorbent for U(VI) displacement.

## 1. Introduction

Nuclear power plants are a sustainable energy source and can produce more energy from uranium than any fossil fuel, including coal and petroleum oil. Furthermore, the increased costs of other renewable energy sources, such as wind, solar, and hydraulic, mean that nuclear energy is a better long-term investment. Their abundant energy resources are being depleted at an alarming rate due to ever-increasing global demand. Wastewater contains heavy metals alongside long-lived radionuclides, which are dangerous pollutants whose migration with groundwater is greatly influenced by adsorption on geological materials [1,2]. The removal of radioactive and toxic metals from wastewater is a major environmental concern [3,4]. Scientists are interested in developing several techniques for the treatment of wastewater [5,6,7]. Generally, wastewater comes from nuclear fuel production and laboratories that handle radioactive substances [8]. Uranium precipitation and removal from its leaner sources is now the focus of research and development on methods and composites that are more effective in the adsorption process [9,10,11,12].

Higher loading capacities can be achieved using adsorbents that allow fast reaction rates. Polymers and fibrous materials that are hydrophilic are excellent adsorbents because of their kinetics [13,14]. It is possible to classify sorbents based on their chemical composition into organic (natural polymers and synthetic polymers), inorganic (silica gel and other oxide species), and natural sorbents, such as white sand and clay, fall under the broad category of sorbents.

Cations are expected to be adsorbed by silica, while the most alkaline oxides (such as MgO) adsorb the anions due to their basic properties. Hydroxyl groups are thought to play a role in the adsorption of ions onto oxide surfaces [15]. The cations are retained when these groups are negatively charged (deprotonated), whereas the anions are retained when they are positively charged (protonated). As a result, on amphoteric oxides (e.g., Al_2_O_3_, TiO_2_, and ZrO_2_), cations are extracted under primary conditions (a pH higher than the oxide isoelectric point, which was noted to be 6.1 of TiO_2_) [16]. On the other hand, anions can only be absorbed in acidic environments (pH below the isoelectric point of the oxide). The use of magnetic Fe_3_O_4_ and SiO_2_ composites for removing U(VI) from solutions was investigated [17]. Using the solid-phase extraction approach, the sodium dodecyl sulfate/alumina/Schiff’s base was used to determine the UO_2_^2+^ ions in the environment [18].

The surfaces of montmorillonite, silica (SiO_2_), and alumina were utilized to extract U(VI) at pH 3.1–6.3. According to these findings, ion exchange occurs during uranyl ion adsorption on montmorillonite at low pH, and the inner-sphere uranyl liquid structure is unaltered. pH values close to neutral and competing cations can lead to an inner-sphere complexation with the surface. According to uranyl adsorption on these surfaces, an inner-sphere, bidentate complexation of uranyl with silica and alumina surfaces appears to occur by forming polynuclear surface complexes with near-neutral pH [19]. Uranium was removed from the water solution and a mixture of aluminum hydroxide, ferric hydroxide, and activated carbon. The uranium extraction from dilute aqueous solutions was investigated using mixed adsorbent at various temperatures and pH levels. In the pH range of 4.0 to 5.5, the adsorbability peaked and then declined as the temperature rose. The best eluting solution was ammonium carbonate [20,21].

Furthermore, amidoxime-modified ordered mesoporous silica was produced and it proved the high uptake of uranium from seawater (57 mg/g). Mostly, mesosilica seems to be an excellent choice for the recovery of uranium from an aqueous solution [22,23,24]. Magnetic chitosan microparticles were functionalized by grafting either amidoxime or hydrazinyl amine onto them. U(VI) and Zr(IV) were recovered from the fluid solutions by testing the sorption characteristics in a pH range of 4.0–5.0 [25], poly sulfonamide/nano-silica composite was utilized to adsorb thorium ions from the sulfate solution [26,27].

Additionally, the U(VI) adsorption behavior on amino/silica SBA-15 was also reported. Many parameters were studied to determine the adsorption properties of U(VI) in discontinuous settings. When the grafted materials were exposed to U(VI), adsorption reached equilibrium in about 30 min, with the most effective material’s extreme U(VI) sorption capacity of 573 mg/g and an ideal pH of 6.0. It has also been discovered that the adsorbed U(VI) is concentrated in the mesopores, generating nanometer-sized U-hydroxy phases [28]. The separation and preconcentration of radioactive uranium using salicylaldehyde/mesoporous silica sorbents were also achieved [29]. In this work, the nano-silica/chitosan (SiO_2_/CS) preparation was described and used to adsorb U(VI) from the definite aqueous phase. The ideal variables for uranium sorption from standard solutions and the wastewater solution are also established, comprising pH, contact time, U(VI) concentration, and temperature.

## 2. Experimentation

### 2.1. Chemicals and Instruments

Analytical-grade chemicals and reagents were employed throughout the operation, with no further purifying in the lab. Merck, Germany, donated the chitin, sulfuric acid, and tetraethyl-orthosilicate used in this experiment. Uranyl sulfate and N-phenyl anthranilic acid were purchased from the B.D.H. lab., England.

Uranium was found using a JENWAY UV/Vis. 6405 spectrophotometer with quartz cells of 1 cm in diameter and a detection wavelength of 655 nm. These changes in the mineralogical composition were investigated using X-ray diffraction (XRD) on Bruker company model D8 (which also included reflection spectroscopy (RF), high-resolution diffraction (HRD), in-plane graining incidence diffraction (IP-GID), residual stress, and texture measurements). IR resolution Software was used to analyze the adsorbents and create samples for FTIR characterization (Shimadzu I.R. Prestige21). An environmental scanning electron microscope (SEM-EDX) (Philips XL 30, Eindhoven, The Netherlands) was used to identify precipitated uranium qualitatively. Additionally, in addition, a quick qualitative investigation of the mineral sample was carried out using this method. Low vacuum and 30 KV were the operating conditions. Model Nova Touch LX2, manufactured by Quantachrome Corporation, Boynton Beach, FL, USA, operated by Windows® Version 1.12, Microsoft, Redmond, WA, USA, has be used to study surface area and pore volume of samples by nitrogen sorption at 77 K.

### 2.2. Chitosan Synthesis

Chitin deacetylation [30,31,32,33] was performed to achieve CS by stirring 20 g of chitin in 100 milliliters of a solution containing 50% sodium hydroxide. The mixture was held at a shallow temperature of −85 °C for 28 hr after being ultra-frozen to that temperature. After that, the mixture’s temperature was increased to 112 °C, and it was agitated for 8 h at a rate of 275 revolutions per minute. The chitosan was filtered, rinsed with deionized H_2_O adjacent to pH 7.0, and then dried in a furnace at 65 °C once it had been obtained.

### 2.3. Nanosilica Synthesis

Hydrothermal synthesis yielded mesoporous, spherical nano-silica [34,35]. The suspension of cetyltrimethylammonium bromide (CTAB) in deionized water, with the addition of 1 M NaOH (10 mg/mL) and an equal volume of water, was carried out at 80 °C. The acceptable solution was then supplemented with 11.7 mL of TEOS and vigorously agitated before being added. The white precipitate was obtained by filtering after two hr of continuous rousing at 80 °C, and it was washed away at least three times with ethanol before drying for 12 h at 60 °C. The surfactant was removed by calcining the powder for 4.5 h at 600 °C, and the resulting fine powder was then tested for nano-silica using a spectrophotometric method.

### 2.4. Silica/Chitosan Synthesis

The sol–gel method was used to synthesize silica/chitosan [36,37]. Two different solutions were made using a unique method. First, 2 mL of water and hydrochloric acid were added to 60 mL of ethanol in the first solution for 10 min, stirring at 250 rpm at 25 °C. A magnetic stirrer was used to slowly add a total of 93 mL of tetraethyl orthosilicate (TEOS) to ensure that the mixture was evenly dispersed. In a rounded bottom flask, 30.0 g of chitosan powder was liquified in 500 mL acetic acid (5.0%) as a second liquid solution. After the chitosan was utterly dissolved in the diluted acetic acid solution, the mixture was agitated for another two hr. The dropwise addition of the first TEOS solution to the second chitosan solution resulted in silica/chitosan. The reactant mixture was agitated for 36 hr at 200 rpm after the solution had been poured in completely. To crosslink the gel, it was allowed to sit for a week before being dried at 55 °C. An airtight bottle labeled “silica/chitosan” held the finished powder.

### 2.5. Uranium Adsorption

This study used the batch approach to adsorb uranium from standard and leaching liquor solutions. U(VI) adsorption was studied by performing a number of experiments in order to determine the optimal values for a number of important factors that influence the sorption process. These variables included pH, contact time, adsorbent dose, and temperature. The mean value of each experiment was used in all cases. Adsorbent dosages of 10 to 100 mg were mixed with 50 mL of uranium solution of various concentrations and shaken at 200 rpm utilizing mechanical shakers for a period of time ranging from 5 to 120 min at various temperatures by ranging the ambient temperature between 25 and 55 °C. NaOH and H_2_SO_4_ solutions adjusted the pH from 1.0 to 6.0. The concentration of metal ions absorbed was determined by comparing the equilibrium and initial concentrations. Different tests were performed on uranium ions to examine their adsorption dynamics, equilibrium isotherms, and thermodynamics by ranging the ambient temperature between 25 and 55 °C.

In each experiment, the adsorption capacity q_e_ (mg/g) and adsorption efficiency (E%), as well as the distribution coefficient (K_d_) of uranium ions on the produced adsorbents, were calculated using the equations below:

(1) $$q_{e}=\left(C_{0}-C_{e}\right)\times \frac{V}{m}$$

(2) $$E\left(\%\right)=\left(\frac{C_{0}-C_{e}}{C_{0}}\right)\times 100$$

(3) $$K_{d}=\left(\frac{C_{0}-C_{e}}{C_{e}}\right)\times \frac{v}{m}$$

This equation provides an easy-to-understand representation for C_0_ and C_e_ as a function of volume (liters, V or milliliters, v), concentration (mg/L), as well as dry adsorbent weight (g).

### 2.6. Uranium Desorption

Desorption was executed on the uranium-loaded SiO_2_/CS gained from the formerly reported set of processes researched to learn more about desorption. This process utilized sulfuric, hydrochloric, nitric acids, sodium carbonate, and ammonium carbonate as eluants. The uranium(VI)-loaded adsorbent was shaken at room temperature with 50 mL of eluent at various concentrations ranging from 0.2 to 1.2 M unless it is stated otherwise. The solid/liquid phase ratio was studied by ranging from 1:10 to 1:70 S/L phase ratio, while the elution time was studied by ranging from 10 to 120 min. The uranium ions were then eluted into the acid solution by filtering the solution. The uranium content was measured by Agilent 7800 ICP–MS, Santa Clara, CA, USA. This study examined the factors that affect uranium desorption, such as the eluent concentration, contact time, and temperature. All of the experiments were triplicated, and the mean value was used.

## 3. Results and Discussion

The newly prepared nano-silica/chitosan (SiO_2_/CS) was characterized and used as an adsorbent for the removal of U(VI) ions from the wastewater solution. Different parameters such as the pH effect, contact time, U(VI) concentration, and temperature have been investigated.

### 3.1. Characteristics

#### 3.1.1. X-ray Diffraction Analysis

**Figure 1:**
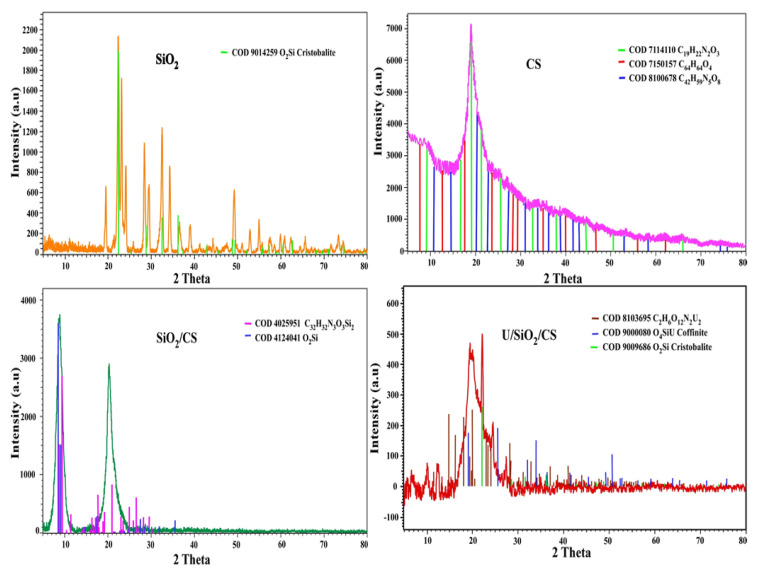
SiO_2_, CS, SiO_2_/CS, and U/SiO_2_/CS XRD patterns.

Figure 1 displays the XRD forms of nano-silica (SiO_2_), chitosan (CS), and nano-silica/chitosan (SiO_2_/CS). These broad SiO_2_ characteristic peaks are matching to Bruker software COD 9014256 database values of 2θ = 19, 22, 24, 24.8, 28. 29, 32, 34, 36, 39, and 49°. Cristobalite was found to make up most of SiO_2_, according to the data gathered from the SiO_2_ pattern. In the database of Bruker software COD 7114110, 7150157, and 8100678, chitosan (CS) revealed a major broad peak at 2θ = 19°. Figure 1 shows the XRD results of SiO_2_/CS and U/SiO_2_/CS. The peak location and peak form were unaffected by the broad peaks of SiO_2_ that overlapped with the XRD pattern of CS, which had a high-intensity peak at 2θ = 19°. In these data, the surface electrostatic interaction between SiO_2_ with CS, which has broad peaks at 2θ = 9 and 20°, besides two little two peaks, appeared at 2θ = 29° and 35°, along with the relevant database of the Bruker program COD 4124041 and 4025951. It was found that some new peaks appeared in the XRD pattern of U/SiO_2_/CS following absorption, according to the Bruker program COD 8103695, 9000080, and 9009686. As opposed to this, there was only a small shift in peak intensities, showing that the composite SiO_2_/CS crystallinity was unaffected by U(VI) adsorption.

#### 3.1.2. EDX and SEM Images

**Figure 2:**
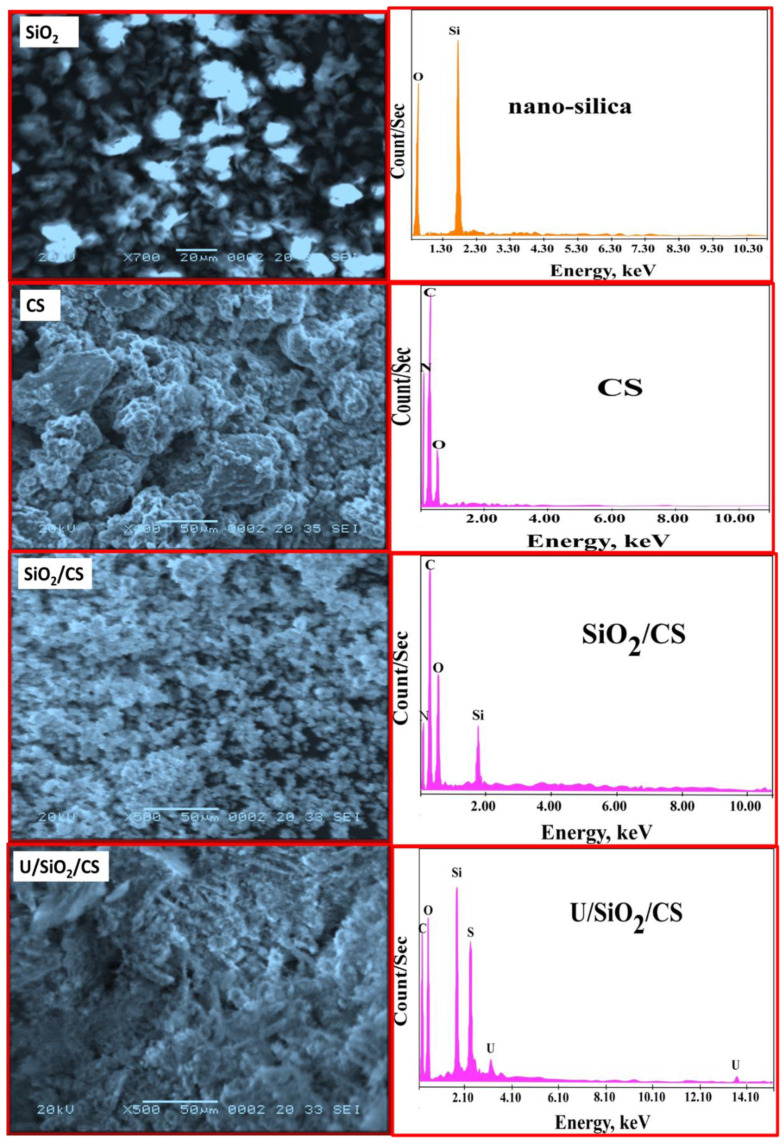
SEM and EDX photographs of SiO_2_, CS, SiO_2_/CS, and U/SiO_2_/CS.

Scanning electron microscopy is the most reliable and convenient tool for examining the physical structure of the resin beads modified in a solvent. In addition, as shown in Figure 2, SEM was used to investigate the surface and physical formations of SiO_2_ and CS. SiO_2_ SEM has revealed distinct shapes and compositions, including distinct roughness. The skeleton of SiO_2_ was built from a variety of small, unrelated pieces with varying diameters. As shown in Figure 2, the surface CS has been smoothed by several holes. Due to the collection and impregnation of small SiO_2_ molecules on the surface of CS, the SEM images show a smooth surface with several cavities. In addition, the composites photographed were made from aggregate particles with larger interstitial holes, as evidenced by the photographs after the SiO_2_/CS was treated with U(VI) adsorption (SEM images revealed that the pores were occupied with U(VI), this composite had erratic surfaces that were agglomerated with U(VI).

Figure 2 illustrates the semi-quantitative analysis of SiO_2_, CS, SiO_2_/CS, and U/SiO_2_/CS. The spectra of SiO_2_ showed just silicon and oxygen peaks; no other peaks could be seen. Additionally, the CS spectrum comprises N, C, and O peaks. C, O, Si, and N peaks were found in the EDX study of SiO_2_/CS. Peaks in Si, N, C, and O can be found in the SiO_2_/CS. There are separate peaks after uranium ion adsorption on SiO_2_/CS. The uranium peaks confirmed uranium (VI) adsorption on SiO_2_/CS.

#### 3.1.3. BET Surface Examination

**Figure 3:**
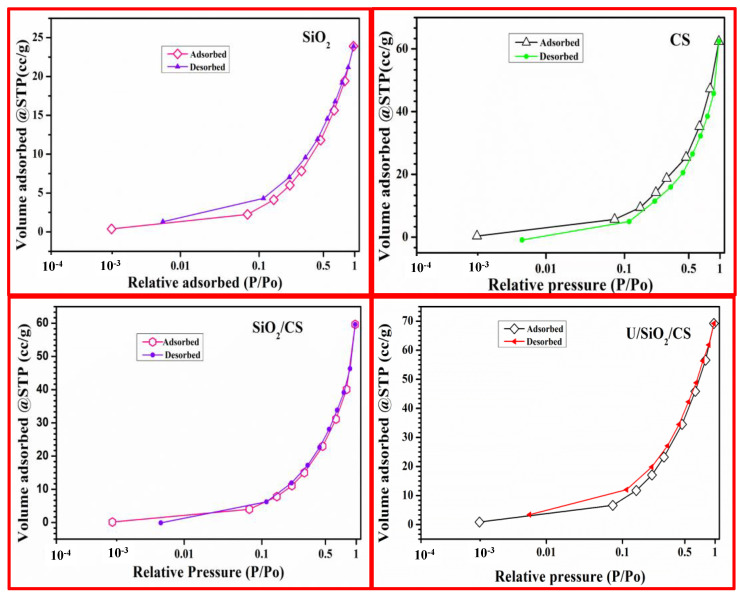
N_2_ adsorption/desorption isotherm of SiO_2_, CS, SiO_2_/CS, and U/SiO_2_/CS.

To determine the surface area, BET was used. Information about the physical structure of the material can be achieved by studying its surface area, which determines how a solid will interact with its environment. Figure 3 presents the N_2_ sorption-desorption isotherm curvatures of the examined ingredients. After the CS was improved with SiO_2_, the isotherms were altered.

Table 1 revealed that the BET surface areas of SiO_2_, CS, SiO_2_/CS, and U/SiO_2_/CS were 25.85, 19.77, 24.55, and 22.71 m^2^/g, respectively. The surface area (S_BET_), pore volume, and pore size of SiO_2_/CS were discernibly different from those of their constituents due to the addition of SiO2 to the CS surface, the surface area has been enhanced, and the uranium ions were better able to adhere to it. This difference may be attributable to the fact that SiO_2_ adorned CS pores. The adsorption of uranium ions adsorption reduced, to some extent, the surface area, pore size, and pore volume. SiO_2_/CS is strongly adsorbable to U(Ⅵ) ions because of the number of active sites.

#### 3.1.4. FTIR Investigation

**Figure 4:**
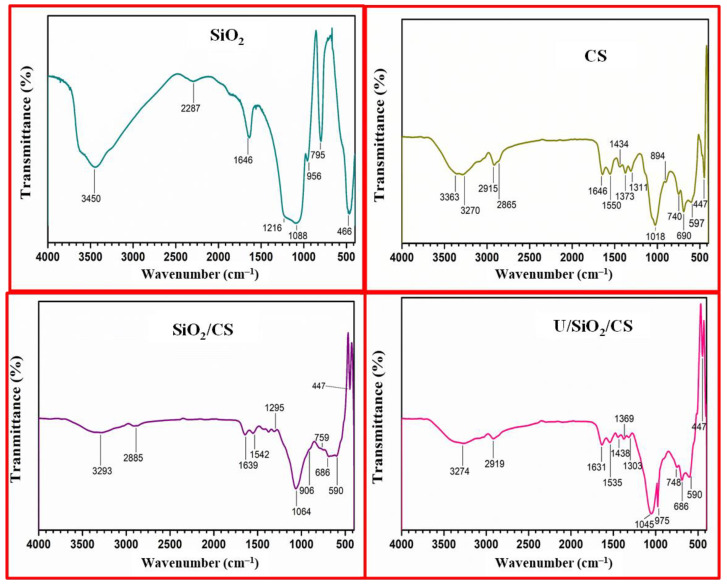
SiO_2_, CS, SiO_2_/CS, and U/SiO_2_/CS FTIR spectra.

FTIR spectroscopy was also exploited to assess the nano-SiO_2_, CS, SiO_2_/CS, and U/SiO_2_/CS, as seen in Figure 4. An extensive band at 3450 cm^−1^ was attributed to SiOH stretching, while a distinctive band of H_2_O on SiO_2_ was observed at 1646 cm^−1^ that was not completely displaced by drying [38]. At 1216 and 1088 cm^−1^, the siloxane molecular group (Si–O–Si) was clearly visible [39]. Furthermore, the silanol group was recorded as having an attendance peak of 956 cm^−1^ (SiOH). The vibrational motion of the Si–O–Si group was responsible for both the peak position at 795 cm^−1^ and the observed peak at 466 cm^−1^ [40].

There was a noticeable FTIR peak around 3200–3550 cm^−1^ because of the overlap of OH and NH groups [41]. The peaks at 2915 cm^−1^ and 2865 cm^−1^ were assigned to –CH_2_ groups. The distinctive peak at 1646 cm^−1^ is related to the NH_2,_ and 1434 cm^−1^ corresponds to the deformation peak of NH [42]. The peaks at 1550 cm^−1^ and 1166 cm^−1^, and 1018 cm^−1^ fit the stretching C–N, respectively [43]. The stretching vibrations of C–O and C–O–C are also responsible for the 1373 and 1311 cm^−1^ peaks. The characteristic absorption of the d-glucose unit was found in the absorption band at 894 cm^−1^ [44]. 

In contrast, the FTIR scale of SiO_2_/CS demonstrated a broad peak (3292 cm^−1^) belonging to the overlapping of OH of SiO_2_ and NH of CS. Peaks at 1295 cm^−1^ and 1064 cm^−1^ pointed to the siloxane groups (Si–O–Si). Additionally, the predicted Si–OH peak at 906 cm^−1^ matched the Si–O–Si group, and the absorption peak at 759 cm^−1^ was found to be a match. The OH and NH stretching vibration bands of the investigated adsorbents were reduced and shifted to a redshift with 5–10 cm^−1^ after U(VI) adsorption, which may be due to the pickup of U(VI) to the surface adsorbents, as shown in the spectra of U/SiO_2_/CS after U(VI) adsorption. In addition, new peaks of (O=U=O) were discovered at 975 and 748 cm^−1^ [45]. Moreover, two weak peaks appeared of U–O near = 490 cm^−1^ [46]. Thus, NH_2_, NH, and –OH, groups are reactants with uranyl cations in this reaction. Accordingly, it realized that the modified CS has the ability to adsorb U(VI) ions.

### 3.2. U(VI) Sorption

#### 3.2.1. Influence of pH

**Figure 5:**
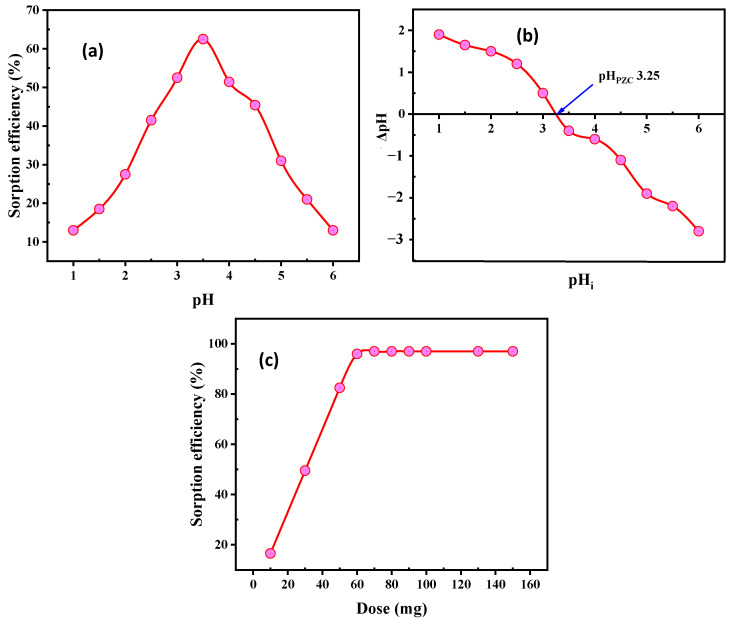
Influence of (**a**) pH, (**b**) pHi and (**c**) SiO_2_/CS dose on the U(VI) sorption exploiting SiO_2_/CS.

The influence of pH on the U(VI) sorption efficacy from the standard solution is exhibited in Figure 5a. Numerous tests were carried out at pH levels ranging from 1.0 to 6.0, with mixed results. This was conducted in conjunction with maintaining the other conditions constant, such as the 50 mL solution concentration, measuring 200 mg/L U(VI), and 30 min contact time at room temperature with a SiO_2_/CS adsorbent dose (0.05 g). It has been found that by increasing the pH from 1.0 to 3.5, the adsorption efficiency of U(VI) ions has consistently increased from 13.0% to 62.5%. It is noteworthy that at high acidity, the functional group of SiO_2_/CS is protonated and competes with uranium anions complexes for adsorption. The condensed uptake was facilitated by the active sites of SiO_2_/CS. Conversely, the adsorption effectiveness was reduced by raising the pH to 6.0 due to the hydrolysis of cationic UO_2_^2+^ and (UO_2_)_2_(OH)_2_^2+^ species to UO_2_(OH)^+^, (UO_2_)_3_(OH)_5_^+^, (UO_2_)_4_(OH)_7_^+^, and UO_2_(OH)_2_ species. Consequently, pH 3.5 is selected as an optimum pH value in the procedures of the subsequent experiments.

The pH at which the net surface charge of the adsorbent is equal to zero is referred to as the pH at the point-of-zero-charge (pH_PZC_). When electrostatic interactions are the major adsorption mechanism, the pH_PZC_ value becomes a crucial metric for interpreting the interactions that take place at the surfaces of materials, particularly for charged adsorbate species. When the pH of the solution is greater than pH_PZC_, the surface of the adsorbent exhibits a negative surface charge due to the adsorption of OH^−^ ions or the deprotonation of hydrogen ions. Under conditions in which pH is lower than pH_PZC_, the surface of the adsorbent exhibits a positive surface charge because of the adsorption of hydrogen ions from the solution [46,47]. It would be very beneficial to identify the point of zero charges (pH_pzc_) of the SiO_2_/CS to calculate the ideal pH for U(VI) sorption. This can be undertaken by determining the value of the pHpzc. For the purpose of deriving the pH_PZC_ value, a plot of the pH change observed after equilibration (pH_f_) and the initial (pH_i_) against the initial pH value (pH_i_) of the solutions was utilized. It was discovered that the pHpzc of SiO_2_/CS was roughly equivalent to 3.25 (Figure 5b). If the pH were less than pHpzc, the SiO_2_/CS would have a positive charge, but if the pH were greater than pH_PZC_, the charge would be negative. Therefore, it is understandable that U(VI) ions are substantially and thermodynamically more practicable to accumulate on the surface at pH values higher than the pH_PZC_ value. Despite this, the efficiency of uranium sorption continues to decline with increasing pH that is greater than pH 3.5. It is because of the progressive creation of the hydroxide precipitate of U(VI). Therefore, it would have a neutral charge at a pH of 3.25 (low charge density), which would demonstrate that the primary mechanism for binding metal ions and their enrichment is complexation with the OH and amino groups. Therefore, a pH of 3.5 is preferable for adsorption purposes.

#### 3.2.2. Influence of SiO_2_/CS Dose

The U(VI) adsorption efficiency was studied under constant conditions of pH 3.5 and 50 mL using a solution containing 200 mg/L U(VI) for 50 min of room temperature contact time in a series of experiments using adsorbent doses of SiO_2_/CS ranging from 10 to 150 mg (Figure 5c). U(VI) adsorption efficiency improved with increasing SiO_2_/CS dose. The adsorption efficiency increased gradually to 60 mg of SiO_2_/CS, remaining constant after that point. As a result, the recommended dose of SiO_2_/CS was 60 mg.

#### 3.2.3. Contact Time

**Figure 6:**
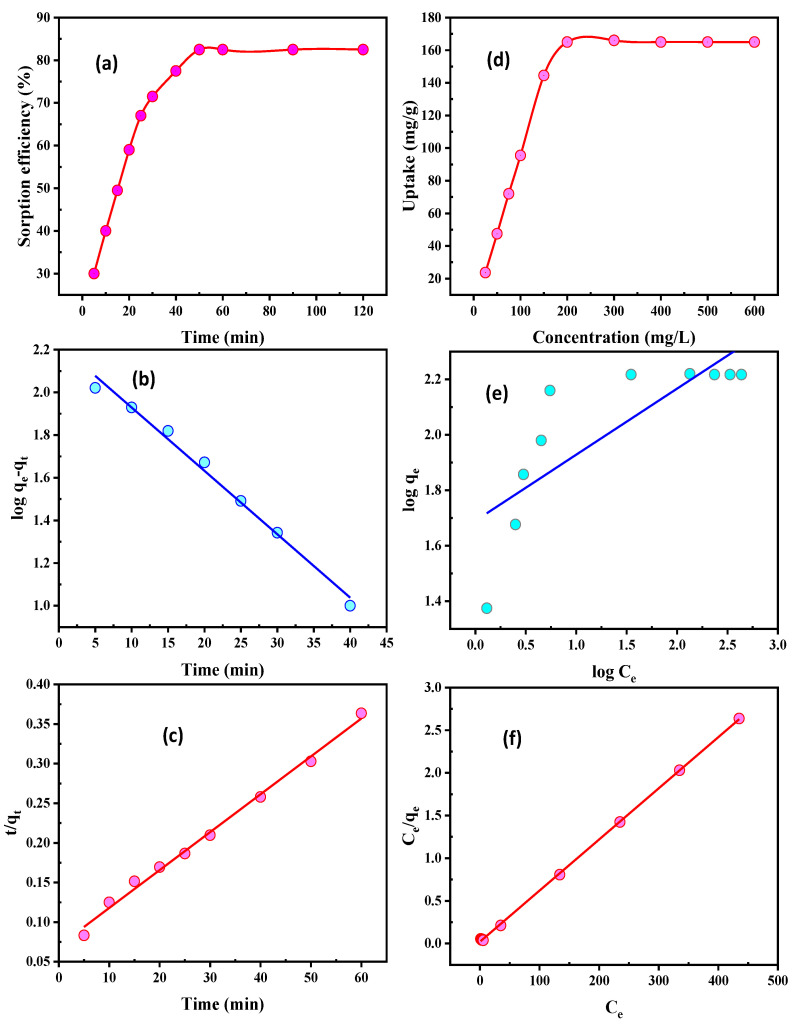
(**a**) Time influence, (**b**) first-order kinetic, (**c)** second-order kinetic, (**d**) U(VI) concentration influence, (**e**) Freundlich isotherm, and (**f**) Langmuir isotherm on U(VI) adsorption using SiO_2_/CS.

The effects of the contact time on SiO_2_/CS U(VI) adsorption were investigated at 5 to 120 min. At room temperature and with SiO_2_/CS doses of 60 mg/L and U(VI) ion concentrations of 200 mg/L, the other adsorption parameters were varied, but all were set to pH 3.5. To reach equilibrium at 50 min (as shown in Figure 6a), U(VI) adsorption improved with increasing contact time. As a result, the minimum time required for additional work was 50 min.

The adsorption processes and rate-controlling steps were studied using kinetic models to determine the mechanism and rate-controlling steps. For the kinetic adsorption mechanism of U(VI) adsorption on SiO_2_/CS, first- and second-order kinetic models were used. The first-order model is given in the linear form, as in the resulting equation [48]:

(4) $$\log\left(q_{e}-q_{t}\right)=\log q_{e}-\left(\frac{k_{1}}{2.303}\right)t$$

The first-order rate constants k_1_ (min^−1^) and q_e_ (mg/g U(VI) at equilibrium and time t (min), respectively, are q_e_ and q_t_. The slope and intercept can be used to calculate k_1_ and q_e_ using the log(q_e_–q_t_) vs. t relationship. The correlation coefficient R^2^ and q_e_ values obtained in Figure 6b show that they do not fit a first-order kinetic model. According to the objective findings, the first-order reaction cannot be carried out using U(VI) adsorption on SiO_2_/CS.

The second-order kinetic model, on the other hand, is implemented and built up in the following equation [49]:

(5) $$\frac{t}{q_{t}}=\frac{1}{k_{2}q_{e}{}^{2}}+\left(\frac{1}{q_{e}}\right)t$$

Here, q_t_ (mg/g) is the amount of U(VI) adsorbed at time t (min), and q_e_ (mg/g) is the amount of U(VI) adsorbed at equilibrium. Chemical adsorption is the rate-dominant step, and this design can predict the kinetic uptake of adsorption. The t/q_t_ vs. t relationship was provided in straight lines when the second-order reaction was valid. The q_e_ and k_2_ were calculated by intercepting and the slope. Figure 6c and Table 2 show a correlation coefficient (R^2^) close to unity, and the calculated value of adsorbed amounts at equilibrium is closer to the practical capacity than the theoretical ones. According to these findings, U(VI) adsorption on SiO_2_/CS was in good accordance with the second-order kinetic.

#### 3.2.4. U(VI) Concentration Influence

This is a crucial parameter of the adsorption technique, and it can affect the adsorption performance of uranium ions. Several batch tests were conducted using 60 mg of SiO2/CS to determine the effect of U(VI) concentration on adsorption efficiency. The standard solution of uranium ions in the range of 25 to 600 mg/L at a pH value of 3.5 was shaken for 50 min at room temperature to conduct these tests. According to Figure 6d, as the uranium ion concentration rose, the adsorption efficiency peaked at 200 mg/L. At 200 mg/L, SiO_2_/CS had the highest adsorption efficiency, at 82.5%. Additionally, the experimental loading capacity of SiO_2_/CS was 165.0 mg/g. Uranium ions completely blocked SiO_2_/CS because the solution’s uranium mobility was the highest.

Adsorption isotherms are effectual systems for the adsorption reaction by ion transfer to the adsorbents [50]. The adsorption isotherms were studied to identify relevant data for adsorption when the adsorbed ions were spread within the solid and aqueous phases when the adsorption process reached equilibrium. The adsorption technique was studied using the models Freundlich and Langmuir. The Freundlich isotherm describes the adsorption of U(VI) on the adsorbent surface [51]. It is commonly utilized to study surface energies and heterogeneity [52,53]. To identify the Freundlich isotherm, the following equation was used:

(6) $$\log q_{e}=\log K_{f}+\frac{1}{n}{\mathrm{logC}}_{e}$$

The amount of U(VI) adsorbed at equilibrium (q_e_) is the amount of U(VI) adsorbed at equilibrium (mg/g), and U(VI) concentration in solution (C_e_) is the constant related to maximum adsorption capacity (K_f_). The regression lines for n and K_f_ were drawn from the logq_e_ vs. logC_e_ curve. The R^2^ value was calculated using data from Table 3 and Figure 6e and was found to be 0.641. Consequently, the results showed that the Freundlich isotherm was not applicable in this case.

Saturated monolayer adsorption on adsorbent surfaces under constant energy conditions is the basis of the Langmuir isotherm model’s ion uptake on a homogenous surface. On the surface, there is no ion association. The following equation quantifies it as follows:

(7) $$\frac{C_{e}}{q_{e}}=\frac{1}{q_{\max}b}+\left(\frac{1}{q_{\max}}\right)C_{e}$$

U(VI) adsorbed/unit mass of adsorbent q_e_ (mg/g) equilibrium, qmax (mg/g) maximum, and b is a constant linked to the affinity of the binding sites and adsorption energy (mg/L). There was a close match between the uptake capacity (166.66 mg/g) and experimental uptake capacity (165.0 mg/g), and the R^2^ was closer to unity. The Langmuir isotherm was clearly observed in U(VI) adsorption. In this work, the uptake capacity is regarded as having achieved a good level of attainment because it has been compared to the data on the uranium uptake provided by other researchers (Table 4).

#### 3.2.5. Temperature and Thermodynamics

**Figure 7:**
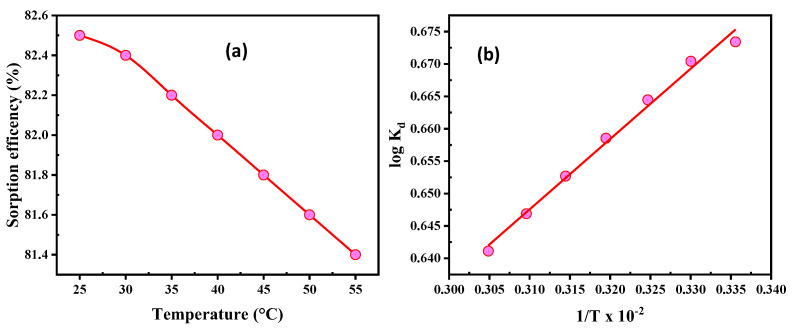
(**a**) Temperature influence, and (**b**) thermodynamic relation for U(VI) adsorption on SiO_2_/CS.

Temperatures ranging from 25 to 55 °C were used to examine the effect of temperature on the adsorption of U(VI). As a result, 50 mL of water was used for the experiments, which were run for 50 min with constant concentrations of 200 mg/L U(VI), pH 3.5, and a SiO_2_/CS dose of 0.05 g (Figure 7a). By raising the temperature to 55 °C, the adsorption rate drops from 82.5 to 81.4%. However, the breakdown of van der Waals bonds leads to a decrease in the number of active sites. As a result, the ideal sorption temperature is found in the ambient air.

The changes in Gibbs free energy (ΔG°), energy enthalpy (ΔH°), and dispersion were measured using adsorption trails at various temperatures for each of the three thermodynamic quantities. The following were determined to be the thermodynamic conditions for U(VI) adsorption [61,62]:

(8) $$\log K_{d}=\frac{\Delta S{}^{\circ}}{2.303R}-\frac{\Delta H{}^{\circ}}{2.303\mathrm{RT}}$$

(9) ΔG°=ΔH°−TΔS°

K_d_ and R are the adsorption equilibrium constant (L/g) and universal gas constant (8.314 J/mol.K), respectively. T stands for the absolute value of a given quantity (K). The negative ΔG° values in Figure 7b and Table 5 indicate that U(VI) adsorption is spontaneous. Because the electrostatic attraction between U(VI) and SiO_2_/CS is strong, the adsorption manners were found to be preferable by analyzing the ΔG° of the interactions. A negative ΔH° may advise exothermic adsorption. ΔS° was negatively biased, indicating that adsorption could occur orderly at the interface between the adsorbent and the solution.

#### 3.2.6. Diverse Ions Effect

The effect of diverse ions on U(VI) sorption was studied by 50 mL of 200 mg/L U(VI), pH 3.5, and 50 mg SiO_2_/CS for a 50 min time of adsorption. Different binary mixtures containing U(VI) ions and the other diverse ions were contacted together under the optimized conditions of U(VI) sorption. The diverse ions were prepared from their salts and added to the original solution. The distribution ratio (D) is calculated from the ratio of metal ions in the solid phase (C_S_) to their concentration in the aqueous phase (C_A_).

D = C_S_/C_A_ (10)

The separation factor (β) indicates the selectivity of the SiO_2_/CS towards U(VI) in the presence of diverse ions.

β = D_U_/D_M_ (11)

where D_U_ and D_M_ are the distribution ratio of uranium and diverse metals, respectively. Furthermore, the tolerance limit is determined and defined as the concentration of diverse ions (mg/L) that cause an error in U(VI) recovery not exceeding ±2%. The possible interference of the associated elements leads to adverse effects on the U(VI) sorption if the diverse metal ions are reacted or sorbed on the SiO_2_/CS active sites. These ions also compete with uranium sorption. From the obtained results in Table 6, it is found that the ions such as Na^+^, K^+^, Ca^2+^, Mg^2+^, Al^3+^, Si^4+^, P^5+,^ and Ba^2+^ do not interfere with 200 mg/L U(VI) to the limit of 2000 mg/L whereas these diverse ions can be tolerated to a greater extent. At the same time, ions such as Ti^4+^, Cr^3+^, Ni^2+^, Cu^2+^, Zn^2+^, Mn^2+^, Zr^4+^, Pb^2+^, V^5+,^ and Fe^3+^ interfere with 200 mg/L U(VI) till a certain limit, whereas these ions do interfere with U(VI) sorption. The results show that SiO_2_/CS is selective in extracting U(VI) from the diverse ions, whereas the separation factor values of SiO_2_/CS are higher.

### 3.3. Adsorption Mechanism

Prior to and following U(VI) adsorption, XRD, SEM, EDX, and FTIR analysis provided valuable information. Figure 1 shows the XRD patterns. The high-intensity CS peak positions and shapes overlapped with the SiO_2_ peak positions. The adsorption of uranium ions resulted in the appearance of new peaks in the XRD pattern of U/SiO_2_/CS. An SEM image of U/SiO_2_/CS (Figure 2) reveals pores filled with U(VI) and a rough, agglomerated surface of U/SiO_2_/CS. The EDX spectra corresponding to the uranium ion sorption were used to identify it (Figure 2). If you look at this graph, uranium is visible at the bottom. Pore-blocking with uranium ions reduced the SiO_2_/CS surface area, pore size, and pore volume, as shown in Figure 3. U(VI) ions were firmly adsorbent on the SiO_2_/CS surface. There were new features of (O=U=O) at 975 and 748 cm^−1^ in the FTIR spectra before and after the adsorption of U(VI). In addition, U-O showed two weak peaks near 490 cm^−1^.

**Figure 8:**
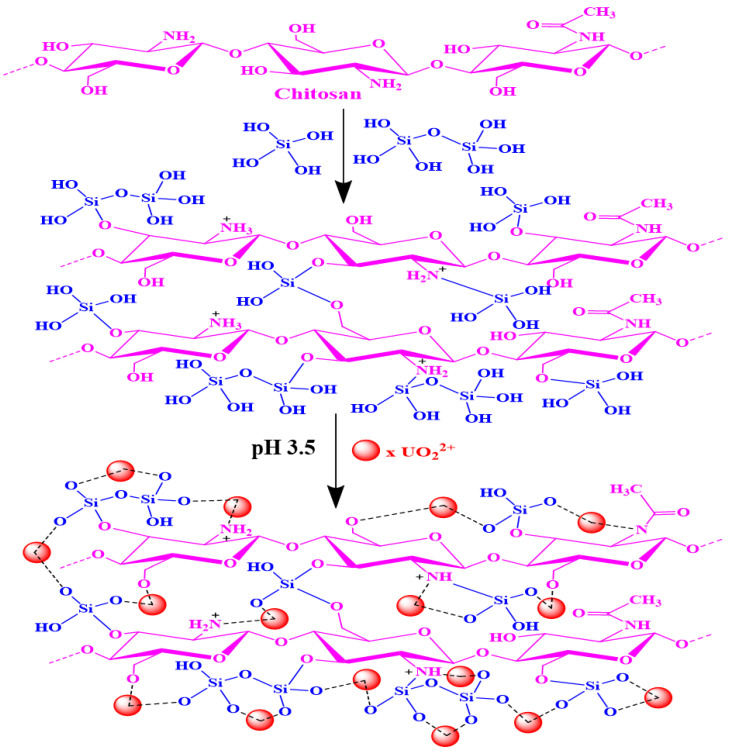
The graphical chart for the adsorption SiO_2_/CS and U(VI) ions.

U(VI) attachment may occur via the deprotonation of functional groups of adsorbents, according to the pH-dependent designation (Figure 5a). The cationic species of U(VI) were found at pH 3.5. The hydroxyl, silanol (Si-OH), NH, and NH_2_ groups on SiO_2_/CS surfaces reacted with uranyl ions at these active sites. Chemisorption was found to control the adsorption mechanism and better fit the second-order kinetic data to the U(VI) adsorption data. The experimental data from the isotherm investigation fit the Langmuir model perfectly. In addition, the adsorption process was exothermic and unforced. Figure 8 depicts one possible mechanism for U(VI) adsorption on the SiO_2_/CS surface.

### 3.4. U(VI) Desorption

Uranium desorption from the uranium-loaded SiO_2_/CS was performed. The desorption manner is genuinely used to reuse and regenerate the adsorbent. In addition, it is a significant aspect of decreasing the adsorbent purification cost. Many aspects affect the desorption efficiency via batch methods, such as eluting concentration, S:L phase ratio, along with desorbing time [63,64,65].

#### 3.4.1. Eluting Type

**Figure 9:**
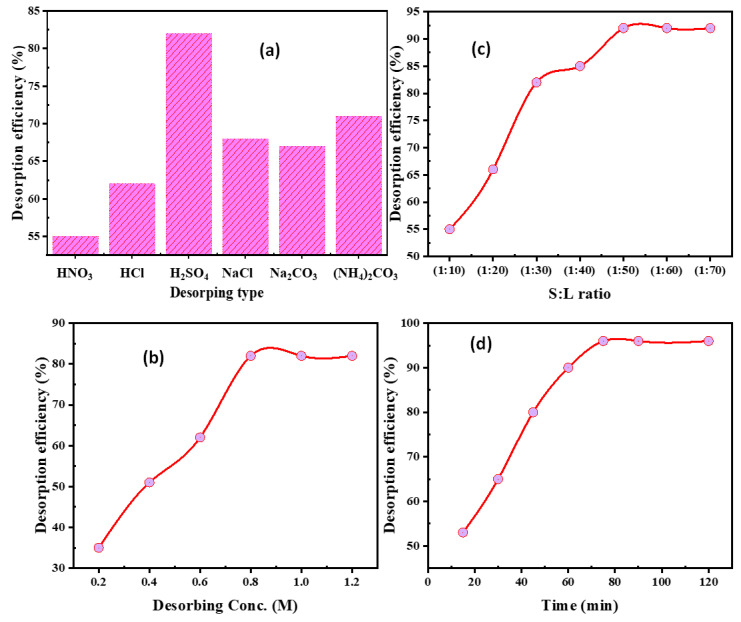
(**a**) Eluting agent, (**b**) H_2_SO_4_ desorbing concentration, (**c**) S:L phase ratio, and (**d**) desorbing time influence upon U(VI) desorption for the U/SiO_2_/CS.

Applying different eluting types, such as HNO_3_, HCl, H_2_SO_4_, NaCl, Na_2_CO_3_, and (NH_4_)_2_CO_3_, affected the elution of U(VI) ions from the uranium-loaded-SiO_2_/CS. Despite this, the additional desorption parameters remained constant over 60 min at room temperature and an elution concentration of 1.0 M at an S:L ratio of 1:30 (30 mL eluent to 1.0 g U/SiO_2_/CS). Figure 9a illustrates that the U(VI) desorption via 1.0 M H_2_SO_4_ attained the maximum desorption at 82.0%. Consequently, it was settled that sulfuric acid was suitable for desorption.

#### 3.4.2. H_2_SO_4_ Concentration

The eluting concentration plays a substantial role in metal ion desorption from the loaded sorbent. Using an H_2_SO_4_ concentration ranging from 0.2 to 1.2 M, the U(VI) desorption from U/SiO_2_/CS was performed. Still, the related parameters remained constant at 1.0 g, S:L phase ratio of 1:30, and 60 min of contact time at room temperature. Figure 9b shows that by increasing the H_2_SO_4_ concentration from 0.2 to 0.8 M, the desorption rate increased to 82.0%. Accordingly, 0.8 M H_2_SO_4_ was chosen for the subsequent desorption procedure.

#### 3.4.3. S:L Phase Ratio

Identifying the most acceptable minimum eluent volume to elute the U(VI) ions from U/SiO_2_/CS is significant. To examine the minimum eluting volume for the U(VI) desorption from U/SiO_2_/CS, various volumes of 0.8 M sulfuric acid were used in the range from 10 to 70 mL, which was added to 1.0 g U/SiO_2_/CS for 60 min desorbing time (Figure 9c). As the S:L phase ratio decreased to 1:50, the data showed that the desorption of uranium ions increased; after this, the U(VI) desorption remained nearly constant at 92.0%. As a result, the following experiments used an S:L ratio U/SiO_2_/CS of 1:50.

#### 3.4.4. Desorbing Time

At room temperature, 50 mL of 0.8 M H_2_SO_4_ was used to stir 1 g U/SiO_2_/CS for contact time ranging from 10 to 120 min. Figure 9d shows that the best time for maximum desorption (96.0%) was 75 min of contact time and remained constant, indicating that the system had reached equilibrium. As a result, 75 min was the optimal desorption time.

### 3.5. Regeneration of the SiO_2_/CS

The reusable U/SiO_2_/CS has undergone regeneration multiple times. Then, 0.8 M of H_2_SO_4_ and a 1:50 S:L ratio were used to regenerate the U/SiO_2_/CS for 75 min at ambient temperature. The sorption–desorption developments were recurrent many times till desorption reduced from 96.0 to 81.0% after seven consecutive series. It designated the good adsorption constancy of the SiO_2_/CS for uranium recovery.

## 4. Conclusions

A simple hydrothermal process can be used to make nano silica/chitosan (SiO_2_/CS). The analyzing methods used to characterize the SiO_2_/CS prepared include those listed above and EDX analysis, BET, and FTIR. At pH 3.5 and 200 mg/L, metal ions, 50 min of sorbing time, and 50 mm of SiO_2_/CS were used to remove U (VI). The data showed that a sorption uptake of 165 mg/g was optimal. The investigation results revealed that the SiO_2_/CS imitated U(VI) adsorption followed a monolayer chemical sorption progression and was consistent with second-order kinetic and Langmuir models. Adsorption on SiO_2_/CS was an exothermic and spontaneous development. To reuse and recycle the SiO_2_/CS, a desorption process using 0.8 M H_2_SO_4_ and a 1:50 S:L phase ratio was carried out for 75 min. It took seven series for the desorption to drop from 96.0 to 81.0 percent, but it eventually stabilized. Using the SiO_2_/CS adsorbent developed in this study, U(VI) adsorption was found to be very effective.

## Figures and Tables

**Table 1:** Surface parameters of SiO_2_, CS, SiO_2_/CS, and U/SiO_2_/CS.

Materials	S_BET_, m^2^/g	Pore Size, nm	Pore Volume, cc/g
SiO_2_	25.85 ± 0.21	2.87 ± 0.08	0.037 ± 0.006
CS	19.77 ± 0.32	2.65 ± 0.09	0.033 ± 0.005
SiO_2_/CS	24.55 ± 0.43	2.75 ± 0.07	0.035 ± 0.007
U/SiO_2_/CS	22.71 ± 0.26	2.59 ± 0.08	0.032 ± 0.008

**Table 2:** For U(VI) adsorption Kinetic parameters of SiO_2_/CS.

Pseudo-1st-Order			Pseudo-2nd-Order		
q_e(cal)_ (mg/g)	k_1_ (1/min)	R^2^	q_e(cal)_ (mg/g)	k_2_ (g/mg.min)	R^2^
106.19	0.068	0.942	181.82	5.69 × 10^−4^	0.995

**Table 3:** U(VI) adsorption isotherm parameters of SiO_2_/CS.

Freundlich			Langmuir		
K_f_ (mg/g)	n (mg.min/g)	R^2^	q_max_	b (L/mg)	R^2^
48.99 ± 2.13	4.196 ± 0.93	0.641	166.66 ± 3.77	0.290 ± 0.04	0.9998

**Table 4:** Comparison of U(VI) uptake capacity of SiO_2_/CS among other adsorbents.

Sorbent	Uptake (mg/g)	References
Free silica gel	21.40	[19]
Nanoporous silica	29.40	[54]
SiO_2_/graphene oxide	145.00	[34]
Triamine modified silica (TAMS)	90.30	[55]
Pentamine modified silica (PAMS)	112.00	[55]
Chitosan	68.0	[56]
ZrO_2_/Chitosan	175.00	[57]
Deacetylated chitosan	17.44	[58]
Amidoxime/chitosan/bentonite	49.09	[59]
Chitosan/attapulgite	53.5	[60]
SiO_2_/CS	165.00	This work

**Table 5:** Thermodynamic U(VI) adsorption settings at SiO_2_/CS.

T, K	298	303	308	313	318	323	328
∆G°, kJ/mol	−0.392 ± 0.05	−0.369 ± 0.08	−0.347 ± 0.09	−0.325 ±0.06	−0.302 ± 0.05	−0.279 ± 0.07	−0.257± 0.06
∆H°, kJ/mol			−1.73 ± 0.09				
∆S°, kJ/(mol. K)			−0.45 × 10^−2^ ± 0.009				
R^2^			0.9915				

**Table 6:** Diverse ions effect on U(VI) sorption efficiency using SiO_2_/CS.

Diverse Ions	Tolerance Limit, W/W *	Adsorption Efficiency, %	Separation Factor, β
K^+^	2000 ± 20.17	99 ±1.89	1 × 10^5^ ± 42
Na^+^	2000 ± 19.34	99 ± 1.93	1 × 10^5^ ± 71
Mg^2+^	2000 ± 21.45	99 ± 2.01	1 × 10^5^ ± 64
Ca^2+^	2000 ± 22.44	99 ± 1.99	1 × 10^5^ ± 55
Al^3+^	2000 ± 18.58	99 ± 1.79	1 × 10^5^ ± 65
Si^4+^	2000 ± 19.77	99 ± 1.94	1 × 10^5^ ± 64
P^5+^	2000 ± 20.78	99 ± 1.97	1 × 10^5^ ± 63
Ba^2+^	2000 ± 21.23	99 ± 1.89	1 × 10^5^ ± 63
V^5+^	400 ± 12.22	99 ± 1.99	1 × 10^5^ ± 58
Zn^2+^	400 ± 12.22	99 ± 1.92	1.6 × 10^5^ ± 59
Ni^2+^	400 ± 11.45	98 ± 1.88	1.6 × 10^4^ ± 47
Mn^2+^	500 ± 10.76	99 ± 1.91	2.5 × 10^4^ ± 45
Cr^3+^	400 ± 9.79	98± 1.89	1.6 × 10^4^ ± 51
Fe^3+^	500 ± 11.23	98 ± 1.98	5 × 10^3^ ± 52
Pb^2+^	300 ± 8.85	98 ± 1.96	1.4 × 10^4^ ± 61
Zr^4+^	300 ± 8.81	98 ± 1.95	8.9 × 10^3^ ± 71
Ti^4+^	500 ± 10.21	99 ± 1.92	2.5 × 10^4^ ± 59
Cu^2+^	350 ± 8.89	99 ± 1.97	1.6 × 10^4^ ± 56

* Weight ratio of the individual interfering ion to that of U(VI).

## Data Availability

Not applicable.
